# One-Year Follow-Up Examination of the Impact of the North Carolina Healthy Food Small Retailer Program on Healthy Food Availability, Purchases, and Consumption

**DOI:** 10.3390/ijerph15122681

**Published:** 2018-11-28

**Authors:** Stephanie B. Jilcott Pitts, Qiang Wu, Kimberly P. Truesdale, Lindsey Haynes-Maslow, Jared T. McGuirt, Alice Ammerman, Ronny Bell, Melissa N. Laska

**Affiliations:** 1 Department of Public Health, Brody School of Medicine, East Carolina University, Greenville, NC 27834, USA; bellr16@ecu.edu; 2 Department of Biostatistics, College of Allied Health Sciences, East Carolina University, Greenville, NC 27834, USA; wuq@ecu.edu; 3 Department of Nutrition, University of North Carolina at Chapel Hill, Chapel Hill, NC 27599, USA; Kim_Truesdale@unc.edu (K.T.); alice_ammerman@unc.edu (A.A.); 4 Department of Agricultural and Human Sciences, North Carolina State University, Raleigh, NC 27695, USA; lhaynes-maslow@ncsu.edu; 5 Department of Nutrition, University of North Carolina at Greensboro, Greensboro, NC 27412, USA; jtmcguir@uncg.edu; 6 Division of Epidemiology and Community Health, University of Minnesota, Minneapolis, MN 55454, USA; nels5024@umn.edu

**Keywords:** nutrition policy, food deserts, health disparities, rural populations

## Abstract

We examined the short-term impact of the North Carolina Healthy Food Small Retailer Program (HFSRP), a legislatively appropriated bill providing funding up to $25,000 to small food retailers for equipment to stock and promote healthier foods, on store-level availability and purchase of healthy foods and beverages, as well as customer dietary patterns, one year post-policy implementation. We evaluated healthy food availability using a validated audit tool, purchases using customer bag-checks, and diet using self-reported questionnaires and skin carotenoid levels, assessed via Veggie Meter™, a non-invasive tool to objectively measure fruit and vegetable consumption. Difference-in-difference analyses were used to examine changes in HFSRP stores versus control stores after 1 year. There were statistically significant improvements in healthy food supply scores (availability), with the Healthy Food Supply HFS score being −0.44 points lower in control stores and 3.13 points higher in HFSRP stores pre/post HFSRP (*p* = 0.04). However, there were no statistically significant changes in purchases or self-reported consumption or skin carotenoids among customers in HFSRP versus control stores. Additional time or other supports for retailers (e.g., marketing and promotional materials) may be needed for HFSRP implementation to influence purchase and consumption.

## 1. Introduction

The high prevalence of obesity in the United States (U.S.) is a major public health concern because obesity is associated with increased risk for type 2 diabetes [1,2], cardiovascular disease [3], and some cancers [4,5]. This high prevalence of obesity could be decreased if the majority of the U.S. population consumed a diet rich in fruits, vegetables, lean protein, whole grains, and healthier beverages [6,7,8,9]. Yet, U.S. residents do not consume adequate quantities of such health-promoting foods and beverages [10]. Additionally, obesity rates are higher [11,12,13] and fruit and vegetable intake is lower [14] in rural areas compared to urban areas. These rural-urban disparities may be partially due to lower access to retail food outlets offering healthier foods and beverages in rural and low-income areas [14,15,16,17].

Healthy corner store initiatives are one way to address the obesity epidemic [18,19] by providing healthier foods and beverages in small retail food outlets where less healthy foods and beverages are often purchased [20,21]. Research findings regarding the health-related impact of healthy small store initiatives are mixed [18,19,22]. Implementation and evaluation of healthy corner store interventions (including technical assistance programs designed to support retailers in offering healthier options) have occurred in several larger, more urban U.S. municipalities, with most showing improvements in outcomes such as availability of healthier foods and beverages [23], customer dietary intentions [24], and with some evidence of customer dietary improvements [18,19,25]. However, in rural areas, small stores may be the only retail food option available [26,27], indicating the critical influence that small stores may have on customers’ accessibility to healthy foods and dietary behaviors.

In spring 2013, in response to concerns about insufficient healthy food access in a specific congressional district, a member of the North Carolina (NC) House of Representatives introduced House Bill (HB) 957, which resulted in the creation of the House Committee on Food Desert Zones. The Committee held four meetings between January and April 2014 and issued recommendations during its final meeting. In spring 2015, legislators filed HB 250, Healthy Food Small Retailer/Corner Store Act and companion bill Senate Bill (SB) 296, with bipartisan support. Neither HB 250 nor SB 296 was voted on, yet on 1 July 2016, the NC Legislature passed budget adjustments, which included $250,000 for the creation of a statewide Healthy Food Small Retailer Program (HFSRP). Each year since 2016, the HFSRP has been funded through an appropriations bill, allocating $250,000 per year ($750,000 total thus far) to be administered through the NC Department of Agriculture & Consumer Services (NCDA&CS) to small food retailers (defined as having less than 3000 square feet) located in food deserts, “for the purchase and installation of refrigeration equipment, display shelving, and other equipment necessary for stocking nutrient-dense foods, including fresh vegetables and fruits, whole grains, nuts, seeds, beans and legumes, low-fat dairy products, lean meats, and seafood” [28]. The impetus behind this legislation was to provide residents of USDA-defined NC food deserts with greater access to healthy foods and beverages, while also benefiting small food retailers financially. In spring 2017 [29] and again in 2018, we collected data to evaluate the HFSRP in regards to store- and customer-level dietary outcomes. The purpose of this study was to examine changes in availability of healthy foods, purchases, and consumption before and after implementation of the HFSRP in four HFSRP stores compared to four control stores not receiving HFSRP funding.

## 2. Materials and Methods

Before HFSRP implementation, in February–May 2017, we collected baseline data in 16 small food retailers before any HFSRP stores received equipment or started stocking healthier foods and beverages: five that received HFSRP funds to install new equipment and stock and promote healthier foods and beverages in 2017, and 11 control matched stores [29]. In February–May 2018, we collected Year 1 follow-up data in four HFSRP stores and four control stores. The current paper describes our Year 1 follow-up evaluation of the NC HFSRP. The new equipment was in the stores approximately six months prior to Year 1 follow-up data collection.

### 2.1. Store Selection

In 2016, six corner stores received HFSRP funding which was provided via a competitive application process. The evaluation team (the authors) did not participate in selecting the HFSRP stores which were selected for funding. We collected baseline data in February–May 2017 in five of the six stores, as the NCDA&CS HFSRP coordinator felt that one store was undergoing too many transitions for our team to collect baseline data in that store. We selected control stores matched to the HFSRP stores based on a variety of factors, including North American Industry Code Standards (NAICS) store type (small grocery or convenience store), store size, census tract food desert type, and similar demographics, including percent of the census tract on Supplemental Nutrition Assistance Program (SNAP) benefits and the percent African American residents. [29] Due to resource constraints, for our one-year follow-up, we collected data in a subset (*n* = 4) of the 11 original control stores and in 4 of the originally-funded HFSRP stores. Table 1 below includes matching variables for each matched pair.

### 2.2. HFSRP Intervention

The details of what each of the four HFSRP stores did using the HFSRP funding are described in Table 2 below.

### 2.3. Healthy Food Availability

This was assessed using the Healthy Food Supply (HFS) score, as described in our previous work [29]. Briefly, in-store audits were conducted using a form adapted from the validated Nutrition Environment Measures Survey for Stores (NEMS-S) [30]. The adapted NEMS-S includes 10 categories including 18 foods/food types. We replicated the method of Andreyeva et al. [31] and Caspi et al. [30] to create a store-level HFS score, summarizing availability, price, quality, and variety of food and beverage items in each store. The HFS score has a possible range of 0–31, with higher scores indicating healthier items [30].

### 2.4. Ethical Issues and Informed Consent

The East Carolina University Institutional Review Board reviewed and approved study #UMCIRB 16-002420 on 31 January 2017. We obtained a waiver of informed consent from the East Carolina University (ECU) Institutional Review Board and thus obtained verbal consent after customers were given written information about the study. Customers were provided with a $10 gift card upon survey completion.

### 2.5. Customer Intercept Survey

We conducted customer intercept surveys in each store both at baseline and Year 1 follow-up. Participants were recruited by interviewers after making purchases. The intercept survey consisted of 45 items which included a “bag check” (described below), frequency of shopping at the store, shopping at other small stores, availability of fresh fruits and vegetables in the neighborhood, fruit, vegetable, and sugary beverage consumption, and demographics (sex, age, race, ethnicity, highest grade of school completed, annual household income, employment), self-reported height and weight, and Veggie Meter™ assessment. Consumption of fruits, vegetables, and sugary beverages and the Veggie Meter™ assessment are described below.

### 2.6. Healthfulness of Food and Beverage Purchases

We calculated an aggregate, store-level Healthy Eating Index from customer “bag checks,” wherein interviewers recorded product name, brand, size, quantity and price paid for each item purchased [32]. We used bag check data instead of receipt data, as many small stores do not commonly provide customers with itemized receipts. We calculated a single aggregated store-level Healthy Eating Index (HEI)-2010 score for each store, with a possible range from 0 to 100. The HEI-2010 is comprised of 12 components and scored per 1000 kcal. We used the aggregated score because some purchases involve a very small number of items and therefore make the HEI less meaningful. The NCI Automated Self-Administered 24-hour recall website (ASA24) was used to determine kilocalories, added sugars, fiber and general nutrient profile of purchases made at HFSRP and control stores. We calculated HEI-2010 scores for purchases using the SAS macros provided by NCI. The HEI-2010 is a valid indicator of whether a diet or food source is consistent with federal dietary guidelines [33,34]. The aggregate HEI was calculated by deriving each of the 12 sub-components according to published guidelines [35,36].

### 2.7. Customer Dietary Intake

Participants self-reported fruit and vegetable intake using a single items for both fruits and vegetables, as in Ortega, et al. [37], i.e., “On a typical day, how many servings of fruits do you eat? (A serving of fruit is like a medium sized apple or a half cup of fresh fruit—this does not include fruit juice)” with responses reported as whole numbers. Respondents also reported fruit and vegetable consumption using the NCI Fruit and Vegetable Screener [38]. The NCI screener includes frequency and amount questions for the following items: 100% juice; fruit (fresh, canned, frozen, no juice); lettuce salad; French fries/fried potatoes; other white potatoes; cooked dried beans; other vegetables; and tomato sauce. Data from the NCI screener were analyzed using the older version of the standard NCI algorithms as in our baseline paper [29]. The questionnaire also included two sugary beverage questions regarding frequency of consumption of regular soda and sweetened fruit drinks adapted from the Behavioral Risk Factor Surveillance System (BRFSS) [39].

In addition to self-reported dietary assessment, we used the validated Veggie Meter™ device [40], which operates via pressure-mediated reflection spectroscopy (RS) [41,42] to assess skin carotenoid status as a proxy for fruit and vegetable consumption. The Veggie Meter™ takes measurements in the fingers and as in our baseline data collection, at follow-up, each participant’s finger was scanned 3 times and the average value was used. Body mass index (BMI, kg/m^2^) was calculated from self-reported height and weight. We corrected for the error in self-reported height and weight using a correction factor [43].

### 2.8. Statistical Analysis

We compared data from customers from the four HFSRP and four control stores on demographics and dietary intake. We used t-tests or chi-squared tests to examine pre/post differences in customer characteristics for both HFSRP and control store customers. Unadjusted difference-in-difference analyses between HFSRP and control customers were conducted using two-way ANOVA or logistic regressions. Change in store-level HFS Scores and aggregate, store-level HEI scores for purchases in the eight stores were assessed using two-sample *t*-tests. Change in customer-level fruit, vegetable, and sugary beverage consumption, as well as skin carotenoids and BMI were assessed using adjusted difference-in-difference models (adjusted for age, race, and sex, with repeated-measures to account for clustering of customers in stores) with propensity scores used when there were significant differences in customer characteristics.

## 3. Results

### 3.1. Customer Characteristics

Table 3 includes differences in customers pre/post HFSRP in both HFSRP and control stores. The mean age across the years was 42.5 years–44.9 years and mean BMI was 27.7–30.5 kg/m^2^.

### 3.2. Healthy Food Supply Scores

There was a significant difference in HFS scores in HFSRP versus control stores. The HFS score was –0.44 points lower in control stores and 3.13 points higher in HFSRP stores pre/post HFSRP (*p* = 0.04). Table 4 below lists the stores, the average 2017 HFS score and 2018 score, and the change in HFS score for each store.

HFS scores in HFSRP Store A increased due to an increase in availability of lower fat milk, a higher price of whole versus lower-fat milk, an increase in availability of fresh fruits and vegetables, and an increase in canned vegetable availability. HFS scores in Store B increased due to increases in frozen fruits and vegetables, canned fruit, and increases in availability of brown rice and whole grain cereals. HFS scores in Store C increased due to a higher ratio of low-fat relative to whole milk, more varieties of frozen vegetables and canned fruit. Finally, HFS scores in Store D increased due to increased varieties of fresh vegetables, and more whole grain bread and tortillas.

### 3.3. Healthy Eating Index Scores

The Table 5 below shows the HEI scores of customer purchases, aggregated at the store level for the HFSRP and control stores, as well as the change in HEI Scores. The mean difference in HEI from baseline to Year 1 for control stores was 0.08, and the mean change for HFSRP stores was 1.19, with a mean difference of 1.11, and the *p*-value for this difference was 0.83. Contrary to our hypotheses, the HEI decreased from baseline to Year 1 in three of the four HFSRP stores, indicating customers were making less healthy purchases post-HFSRP intervention, though these differences were not statistically significant.

### 3.4. Fruit, Vegetable and Sugary Beverage Purchases and Intake, Skin Carotenoids and BMI in HFSRP versus Control Stores

There were no significant differences in fruit and vegetable intake, sugary beverage intake, or in skin carotenoids and BMI among customers from HFSRP versus control stores. (See Table 6 below) These results remained similar when income was included as a covariate, so to improve model parsimony, we left income out of the model. 

## 4. Discussion

The HFSRP has the potential to positively improve access to healthy foods and beverages, and thereby, purchases and dietary intake, which could help diminish health disparities among residents of NC food deserts. Despite this potential, for three consecutive years, the HFSRP has lacked the votes to become more permanent in the form of a law (it has only been approved in appropriations bills which are less permanent forms of law and must be passed annually). If there are positive impacts of the HFSRP on dietary outcomes and rural economies, the NC Legislature would have more evidence to pass the bill into a law, versus an annual appropriations bill.

In our current one-year follow-up study, we found that the supply of healthy foods increased in HFSRP versus control stores, demonstrating that the HFSRP is having a positive impact on foods offered in small stores in NC food deserts. We found that HFS scores increased due to increased lower-fat milk offerings, increase in the varieties of fresh, frozen and canned fruits and vegetables, and more whole grain products. Thus, the HFSRP is reaching the goal of providing healthier options to residents who might otherwise not have such options [15,16,17]. However, we did not find any differences in purchases or either self-reported or objectively-measured dietary behaviors among HFSRP versus control store customers. While we did see positive effects on the healthy food supply within HFSRP stores, it could take longer for positive impacts on purchases and diet to occur among customers. Over time, the Special Supplemental Nutrition Program for Women, Infants, and Children (WIC) policy changes in 2009 have resulted in positive changes to the food environment and dietary behaviors among WIC participants [44,45,46,47,48]. The positive changes in the food environment in WIC vendors, in addition to positive changes in dietary behaviors among WIC participants make it reasonable to expect that the HFSRP, which was found to have positive impacts on the availability of healthy foods and beverages, could have similar positive impacts over time.

Caspi et al. [32] found a mean HEI-2010 score across stores in Minneapolis and St. Paul, Minnesota of 36.5. The mean HEI-2010 in NC HFSRP and control stores ranged from 23.5 to 44.7, and HEI-2010 for purchases increased more in the HFSRP stores than in control stores, yet this increase was not statistically significant. Although we did observe negative differences in HEI of purchases in three intervention stores, the change in HEI was not statistically significant. Overall the magnitude of these non-significant changes was also small (HEI changes ranging from 2.22 to 6.76 on a scale of 0–100). It will be interesting to see how the HEI of purchases changes in the coming years, as more HFSRP stores are funded.

In this one-year follow-up study, we did not find any associations between whether a retailer received HFSRP funds and purchased/installed equipment, and customers’ dietary intake. Thorndike et al. [49] conducted a pilot study among three intervention and three control corner stores, to examine if increasing the visibility and quality of fresh produce in corner stores would result in increased fruit and vegetable purchases, and found that this increase in purchases did occur when examining WIC fruit and vegetable voucher redemption (*p* = 0.036) yet the increase was not statistically significant when comparing self-reported fruit and vegetable purchases from baseline and intervention-period exit interview responses among WIC customers. Based on their results, Thorndike et al. [49] conclude that policies that incentivize stores to stock and display high quality produce could promote healthier food choices, again supporting the need for policies such as the NC HFSRP.

The current study was limited by the small sample size of stores, which may limit ability to detect significant effects. Also, due to resource constraints, we conducted the audit and calculated the HFS in one HFSRP store in August 2018 (versus Spring 2018). Thus, the mean for the HFS score among HFSRP stores may be inflated due to increased time for implementation. Another limitation is that the stores were not randomly assigned to HFSRP or control conditions. However, we matched the stores based on store-level factors such as store type and size. Study strengths include objective measures of healthy food and beverage availability, purchases, and diet (fruit and vegetable intake). The study was also set in an underserved and understudied area: rural NC, where interventions of this type are greatly needed.

## 5. Conclusions

There were statistically significant improvements in healthy food supply scores, but there were no statistically significant changes in HEI of purchases or self-reported consumption, skin carotenoids, or BMI among customers in HFSRP versus control stores. It may be that more time or more intensive education and marketing are needed before positive impacts on customer purchase and consumption are evidenced. In the future, it would be beneficial to collect qualitative data to learn more about the successfulness of strategies each HFSRP store tried, and to possibly link these data with changes in customer purchases and consumption.

## Figures and Tables

**Table 1:** Store matching variables for each matched pair of stores included. SNAP: Supplemental Nutrition Assistance Program; NAICS: North American Industry Code Standards.

Store Matching Group	NAICS Designated Store Type	Store Size	Census Tract-Level Food Desert Type	Census Tract % SNAP	Census Tract % African American
Intervention Store A	✓	✓	✓	15%	46%
Control Store A				17%	39%
Intervention Store B	✓	✓	✓	26%	38%
Control Store B				53%	70%
Intervention Store C	✓	✓	✓	21%	44%
Control Store C				38%	81%
Intervention Store D	✓	✓	✓	16%	23%
Control Store D				26%	88%

**Table 2:** Healthy Food Small Retailer Program (HFSRP) stores and details on the intervention. NC: North Carolina.

Intervention Store	HFSRP Details on the Intervention
A	Ordered new equipment and a large promotional event was planned upon installation. However, equipment was not installed at the time of the first report to the NC Legislature on 1 October 2017.
B	Ordered and installed a small freezer (August–October 2017) and converted a candy rack into a produce display. The store owner prepared sliced cucumbers for grab-and-go snacks and sold all that were prepared.
C	Ordered equipment in August 2017 and is partnering with the local health department on promotions to highlight healthier options.
D	Ordered equipment in August 2017, and the owner is now able to stock produce from local farmers.

**Table 3:** Baseline (*n* = 279) and follow-up (*n* = 223) characteristics of customers. BMI: body mass index.

Characteristics	Pre-HFSRP	Post-HFSRP	Pre-Control	Post-Control	*p*-Value for DID ***
Age, years	43.9 (15.3)	44.9 (13.9)	43.8 (14.2)	42.5 (14.8)	0.38
BMI, kilogram/m^2^	30.5 (6.9)	30.1 (6.3)	30.2 (8.6)	27.7 (6.4) **	0.13
% Black	39.5	49.5	87.2	82.3	0.07
% Female	44.2	30.3 *	41.6	40.2	0.15
Daily Fruit Servings	2.08 (2.56)	2.13 (2.87)	1.86 (1.91)	1.98 (2.51)	0.88
Daily Vegetable Servings	2.56 (2.16)	2.43 (1.89)	2.75 (2.26)	2.19 (1.84) *	0.26
Daily Fruit and Vegetables Servings	4.65 (3.76)	4.56 (3.53)	4.59 (3.30)	4.17 (3.24)	0.60
Sugary Beverage Frequency	1.66 (1.95)	1.56 (2.02)	2.22 (2.35)	2.17 (2.30)	0.92
Skin Carotenoids	229.5 (71.7)	236.8 (81.1)	240.5 (99.7)	248.5 (78.0)	0.96

* *p* < 0.05; ** *p* < 0.01, *** DID = difference-in- difference.

**Table 4:** Healthy Food Supply (HFS) scores in 2017 and 2018 in four HFSRP stores and four control stores.

Store	HFSRP or Control	2017 HFS Score (Average)	2018 HFS Score	Change in HFS Score
A	HFSRP	4.00	6.5	2.50
B	HFSRP	4.25	9.5	5.25
C	HFSRP	5.75	6.5	0.75
D	HFSRP	4.00	8.0	4.00
E	Control	5.25	5.5	0.25
F	Control	7.75	5.0	−2.75
G	Control	0.75	2.5	1.75
H	Control	7.50	6.5	−1.00

**Table 5:** Healthy Eating Index (HEI) scores for control and HFSRP stores in 2017 and 2018.

Store	HFSRP or Control	HEI 2017	HEI 2018	HEI Difference
A	HFSRP Store	44.73	40.92	−3.80
B	HFSRP Store	33.60	31.39	−2.22
C	HFSRP Store	36.47	44.50	8.03
D	HFSRP Store	42.31	35.55	−6.76
E	Control Store	33.93	42.35	8.42
F	Control Store	43.75	41.65	−2.10
G	Control Store	33.08	23.46	−9.62
H	Control Store	37.58	40.57	2.99

**Table 6:** Association between dietary outcomes of interest and HFSRP participation after 1 year of follow-up.

Outcome	Parameter Estimate	Standard Error	*p*-Value
Daily servings of fruits and vegetables	0.68	0.64	0.29
Servings of sugary beverages	−0.25	0.37	0.50
Skin carotenoids	−7.92	14.77	0.59
BMI (kg/m^2^)	0.67	1.27	0.60
